# Role of bicarbonate as a pH buffer and electron sink in microbial dechlorination of chloroethenes

**DOI:** 10.1186/1475-2859-11-128

**Published:** 2012-09-13

**Authors:** Anca G Delgado, Prathap Parameswaran, Devyn Fajardo-Williams, Rolf U Halden, Rosa Krajmalnik-Brown

**Affiliations:** 1 Swette Center for Environmental Biotechnology, Biodesign Institute, Arizona State University, PO Box 875001, Tempe, AZ 85287-5701, USA; 2 School of Life Sciences, Arizona State University, Tempe, USA; 3 Ira A Fulton Schools of Engineering, Arizona State University, Tempe, USA

**Keywords:** Acetogen, Alkalinity, Bicarbonate competition, *Dehalococcoides*, pH range, Trichloroethylene

## Abstract

**Background:**

Buffering to achieve pH control is crucial for successful trichloroethene (TCE) anaerobic bioremediation. Bicarbonate (HCO_3_^−^) is the natural buffer in groundwater and the buffer of choice in the laboratory and at contaminated sites undergoing biological treatment with organohalide respiring microorganisms. However, HCO_3_^−^ also serves as the electron acceptor for hydrogenotrophic methanogens and hydrogenotrophic homoacetogens, two microbial groups competing with organohalide respirers for hydrogen (H_2_). We studied the effect of HCO_3_^−^ as a buffering agent and the effect of HCO_3_^−^-consuming reactions in a range of concentrations (2.5-30 mM) with an initial pH of 7.5 in H_2_-fed TCE reductively dechlorinating communities containing *Dehalococcoides*, hydrogenotrophic methanogens, and hydrogenotrophic homoacetogens.

**Results:**

Rate differences in TCE dechlorination were observed as a result of added varying HCO_3_^−^ concentrations due to H_2_-fed electrons channeled towards methanogenesis and homoacetogenesis and pH increases (up to 8.7) from biological HCO_3_^−^ consumption. Significantly faster dechlorination rates were noted at all HCO_3_^−^ concentrations tested when the pH buffering was improved by providing 4-(2-hydroxyethyl)-1-piperazineethanesulfonic acid (HEPES) as an additional buffer. Electron balances and quantitative PCR revealed that methanogenesis was the main electron sink when the initial HCO_3_^−^ concentrations were 2.5 and 5 mM, while homoacetogenesis was the dominant process and sink when 10 and 30 mM HCO_3_^−^ were provided initially.

**Conclusions:**

Our study reveals that HCO_3_^−^ is an important variable for bioremediation of chloroethenes as it has a prominent role as an electron acceptor for methanogenesis and homoacetogenesis. It also illustrates the changes in rates and extent of reductive dechlorination resulting from the combined effect of electron donor competition stimulated by HCO_3_^−^ and the changes in pH exerted by methanogens and homoacetogens.

## Background

Organohalide respiring microorganisms represent a unique, efficient, and sustainable approach to detoxifying chloroethenes contamination from soil, water, and groundwater [1-3]. These microbes are important because they can use priority pollutants such as trichloroethene (TCE), dichloroethene (DCE), and vinyl chloride (VC) as electron acceptors for energy metabolism [4]. *Dehalococcoides* bacteria hold a prominent role among the organohalide respirers isolated to date, as these are the only ones having the proven ability to detoxify chloroethenes to the innocuous end product, ethene [1,5]. *Dehalococcoides* have a constrained metabolism; they strictly utilize hydrogen (H_2_) as the electron donor and acetate as the carbon source [6]. The most common method for delivery of H_2_ and acetate at bioremediation sites is by addition of fermentable substrates as precursors [2,7,8]. H_2_ gas has also been supplied for groundwater field applications [9] and in engineered *ex situ* treatment technologies for chloroethenes remediation [10-12]. In systems fed with H_2_, the pH tends to rise as a result of competing biological reactions, whereas dechlorination and/or fermentation of H_2_-releasing compounds decrease the pH. As a consequence, buffering and pH management are important parameters for assessing *in situ* and *ex situ* remediation approaches, and are crucial for sustained dechlorination [12-14].

In groundwater, dissolution of carbonate (CO_3_^2−^)-containing minerals serves as the natural pH buffer. Among the CO_3_^2−^ species, bicarbonate^a^ (HCO_3_^−^) is the most abundant at neutral pH, and it contributes substantially to the alkalinity of groundwater. Typical HCO_3_^−^ concentrations in groundwater are in the range of 0.7-10 mM [15,16]. Additionally, HCO_3_^−^ is supplemented to groundwater as a common strategy when biostimulation or bioaugmentation are employed in order to buffer the protons produced by the biological reactions [2,8].

In laboratory settings, pH management is also commonly achieved through the addition of HCO_3_^−^ buffer in the form of NaHCO_3_ or KHCO_3_. HCO_3_^−^ has been used for growth of *Dehalococcoides* strains [17] and for mixed dechlorinating communities [18-20] to maintain a favorable pH. *Dehalococcoides* optimum pH has been reported to range from 6.9-7.5 [6]; yet, to date, there is a lack of systematic studies defining both the pH boundaries for these important organisms, and the effect pH exerts on each step in the TCE reduction pathway. Beyond its function as a buffer, HCO_3_^−^ also serves as an electron acceptor for other microorganisms commonly encountered with organohalide respirers in the environment and in laboratory-cultured consortia. For example, at neutral pH, hydrogenotrophic methanogens consume HCO_3_^−^ and H_2_ to generate methane [21]:

Hydrogenotrophic methanogenesis:

(1) $${{\text{HCO}}_{3}}^{-}\;+\;4\;{\text{H}}_{2}\;+\;{\text{H}}^{+}\;\to \;{\text{CH}}_{4}\;+\;{3\;\text{H}}_{2}\text{O}$$

The competition for H_2_ among organohalide respirers and methanogens has been well documented [22-28]. However, none of these studies have addressed how consumption of H_2_, whether added as gas or as a result of fermentation, is affected by varying HCO_3_^−^ concentrations.

Homoacetogens are other important microorganisms commonly encountered with organohalide respirers. Homoacetogens produce H_2_ from fermentation of complex substrates and/or consume available H_2_[29,30]. Hydrogenotrophic homoacetogens catalyze the formation of acetate from H_2_ and HCO_3_^−^ in their energy metabolism [29]:

Hydrogenotrophic homoacetogenesis:

(2) $${{2\;\text{HCO}}_{3}}^{-}\;+\;4\;{\text{H}}_{2}\;+\;{\text{H}}^{+}\;\to \;{\text{CH}}_{3}{\text{COO}}^{-}\;+\;{4\;\text{H}}_{2}\text{O}$$

They, too, compete for H_2_ with organohalide respirers. To date, the limited number of studies that have investigated hydrogenotrophic homoacetogenesis in TCE dechlorinating consortia [27,31] has not included the HCO_3_^−^ concentration as a variable driving the extent and the rates of reductive dechlorination.

Hydrogenotrophic methanogens and homoacetogens can also affect pH in dechlorinating communities. Methanogens produce methane as the end product (Equation 1) by expending one proton and one HCO_3_^−^, while hydrogenotrophic homoacetogens generate acetate (Equation 2) from one proton and two HCO_3_^−^. Both reactions increase the pH while consuming HCO_3_^−^, which often is the only buffer in the system. The effect of HCO_3_^−^ concentration in TCE dechlorinating microbial communities has not been studied. Few studies focusing on other dechlorinating systems have recognized its importance and examined the effect of HCO_3_^−^ concentrations on the formation of chlorinated daughter products, thus motivating this work. For example, removal of chlorophenols from simulated wastewater in upflow anaerobic sludge blanket (UASB) reactors revealed significant inhibition on dechlorination at high HCO_3_^−^ (3500 mg L^−1^ as CaCO_3_) and high pH [32]. In microcosms showing microbial dechlorination of polychlorinated biphenyls with H_2_ gas as electron donor, 100 mg L^−1^ HCO_3_^−^ (1.64 mM) yielded the fastest rates of dechlorination, whereas addition of 1000 mg L^−1^ HCO_3_^−^ (16.4 mM) resulted in the slowest polychlorinated biphenyls dechlorination rates and triggered the most acetate to form [33].

In this study, we evaluate the role of HCO_3_^−^ as a buffering agent and as an electron acceptor in TCE reductively dechlorinating mixed communities using a previously described culture, DehaloR^2, as a model consortium [20]. H_2_, and not fermentable substrates, was used as the sole electron donor to directly and accurately measure hydrogenotrophic production of methane and acetate from HCO_3_^−^. The concentrations of HCO_3_^−^ tested reflect typical groundwater concentrations (2.5-10 mM), as well as commonly reported laboratory concentrations (30 mM).

## Results and discussion

### Chloroethenes reductive dechlorination at different HCO_3_^−^ concentrations

**Figure 1:**
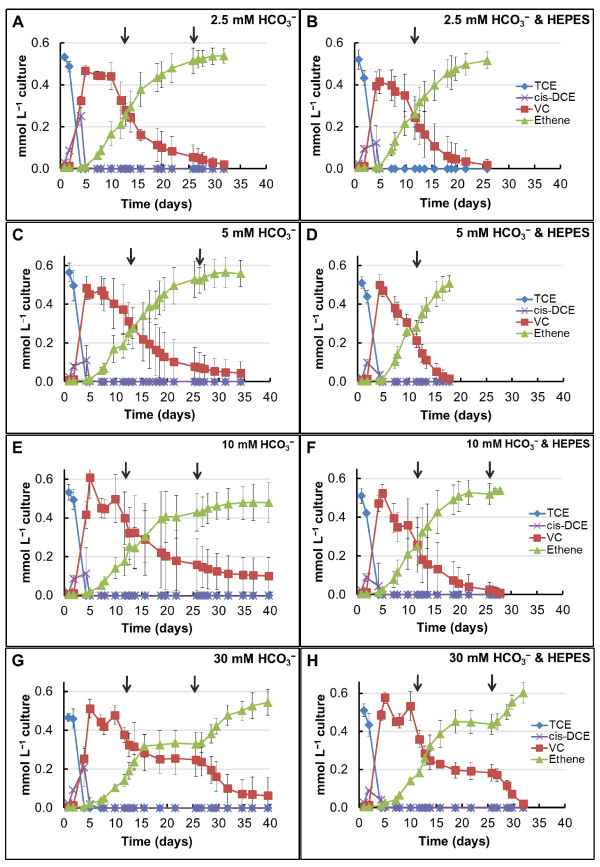
**Chloroethenes dechlorination at different HCO**_**3**_^**− **^**concentrations.** Time course of chloroethenes dechlorination to ethene in cultures amended with H_2_ as the sole electron donor and with HCO_3_^−^ buffer (graphs A, C, E, and G) and a combination of HCO_3_^−^ and HEPES buffers (graphs B, D, F, and H). The arrows represent the 2^nd^ and 3^rd^ addition of 8.2 mmol L^−1^ H_2_. The error bars are standard deviations of triplicate cultures.

The time course dechlorination measurements presented in Figure 1 show a short lag time for the onset of dechlorination of 0.55 mmol L^−1^ TCE. TCE to *cis*-DCE conversion was the fastest dechlorination step in all cultures, with only VC and ethene detected after day 5, regardless of the concentration of HCO_3_^−^ added. A close monitoring of VC to ethene reduction rates between each GC measurement revealed that after day 5, dechlorination rates had slowed down at all HCO_3_^−^ concentrations, especially in the cultures containing 30 mM (Figure 1G-H), suggesting an electron donor limitation. The measured H_2_ levels on day 12 were 1.5 mmol L^–1^ at 2.5 mM HCO_3_^−^ and 0.5 mmol L^–1^ at 5 mM HCO_3_^−^. At 10 and 30 mM HCO_3_^−^, no H_2_ peak was detected on the GC-TCD on day 12. Immediately after injecting an additional 8.2 mmol L^–1^ H_2_ on day 12, we observed an increase in the rates of VC consumption and ethene formation (Figure 1A-H).

Following the second addition of H_2_, all cultures reached ≥70% conversion of TCE to ethene. Complete TCE dechlorination (Figure 1D) was first observed between days 17 and 18 in cultures containing 5 mM HCO_3_^−^ and 5 mM 4-(2-hydroxyethyl)-1-piperazineethanesulfonic acid (HEPES), which was provided as an additional buffer. Complete conversion to ethene was further observed in the cultures with 2.5 mM HCO_3_^−^ & HEPES on day 26. A threefold increase in the 16S rRNA *Dehalococcoides* genes (data not shown) from 1.13 × 10^11^ (±4.98 × 10^9^) copies L^−1^ (time 0) to 3.71 × 10^11^ (±2.78 × 10^10^) and 3.67 × 10^11^ (±8.04 × 10^9^) copies L^−1^ was detected after complete dechlorination at 5 mM HCO_3_^−^ & HEPES and 2.5 mM HCO_3_^−^ & HEPES, respectively. Chloroethenes conversion rates in the cultures containing 10 and 30 mM HCO_3_^−^ were the slowest, as seen in Figure 1. The *Dehalococcoides* 16S rRNA gene copies per L in the cultures with HCO_3_^−^ and HEPES after complete conversion to ethene were 2.07 × 10^11^ (± 5.79 × 10^9^) at 10 mM and 2.03 × 10^11^ (± 5.59 × 10^9^) at 30 mM (data not shown). The lower resulting cell density coupled to decreased dechlorination rates indicates that *Dehalococcoides* growth was diminished at the higher HCO_3_^−^ concentrations (Student’s *t* test; ≥70% confidence level).

We observed a second H_2_ limitation at 10 and 30 mM HCO_3_^−^, with the complete cessation of VC reduction at 30 mM between days 18 and 26 (Figure 1G-H). Consequently, an additional dose of H_2_ (8.2 mmol L^–1^) was injected into all cultures still undergoing dechlorination. With the 3^rd^ addition of electron donor, the 10 and 30 mM HCO_3_^−^ cultures supplemented with HEPES dechlorinated all TCE to ethene by day 28 and 32 (Figure 1F and H), respectively. The parallels without HEPES showed incomplete conversion to ethene even by day 40 (Figure 1E and G) and VC dechlorination had stalled once again on day 35, or it was proceeding at very reduced rates.

### Methane and acetate production during TCE reductive dechlorination

**Figure 2:**
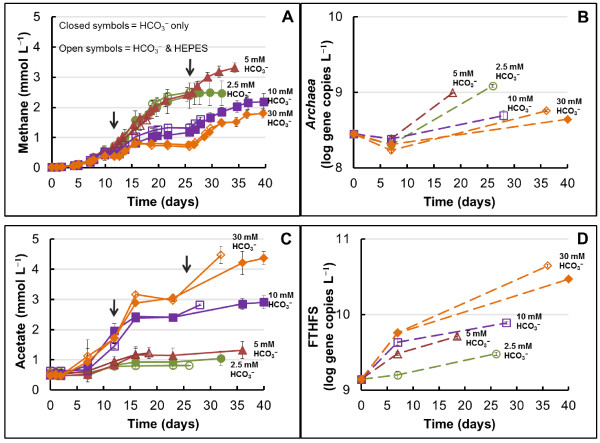
**Methanogenesis and homoacetogenesis during active evolvement of reductive dechlorination.** Methane (**A**) and acetate (**C**) production during reductive dechlorination in medium buffered with 2.5 (circles), 5 (triangles), 10 (squares), and 30 (diamonds) mM HCO_3_^−^. The error bars are standard deviations of triplicate cultures and the arrows represent the 2^nd^ and 3^rd^ addition of 8.2 mmol L^−1^ H_2_. Quantification of methanogens, *Archaea* (**B**), and homoacetogens, FTHFS (**D**) using qPCR. The error bars are standard deviations of triplicate analytical runs.

In Figure 1, we show how H_2_ was limiting dechlorination rates before the 2^nd^ and 3^rd^ H_2_ addition at the different concentrations of HCO_3_^−^ tested. The theoretical H_2_ demand for 0.55 mmol L^−1^ TCE is 1.65 mmol L^−1^H_2_. Considering that the H_2_ at time 0 was 8.2 mmol L^−1^, five times in excess of the theoretical demand for dechlorination, the slower dechlorination rates observed, together with H_2_ depletion, indicated that competing microorganisms were consuming H_2_ faster than the dechlorinators. An increase in methane of only 0.01 mmol L^−1^ was detected at all HCO_3_^−^ concentrations before day 4 (Figure 2A), which coincides with the disappearance of TCE and formation of less chlorinated daughter products (Figure 1). The lack of methane production was also confirmed by the qPCR data which show no relative increase in the numbers of *Archaea* gene copies L^−1^ at this time point compared to the 10% inoculum (Figure 2B, day 7). The lag time for methane production could have been due to the previously reported longer lag time of the methanogenic microorganisms [34] and the toxic effect of TCE on methanogens [31]. Additionally, besides *Dehalococcoides*, other dechlorinators can use TCE as electron acceptor and H_2_ as electron donor to produce *cis*-DCE. A competitive advantage of *Geobacter* spp*.*, the other identified TCE to *cis-*DCE respirers in the inoculum culture [20], over methanogens could have also contributed to a delayed onset of methanogenesis.

Methanogenesis was mostly stimulated at 2.5 mM HCO_3_^−^ and 5 mM HCO_3_^−^, and it was less active with increasing concentrations of HCO_3_^−^ (Figure 2A). The methane production trends observed are supported by a higher increase in *Archaea* numbers at the lower HCO_3_^−^ concentrations (2.5 and 5 mM in Figure 2B) compared to 10 and 30 mM (Figure 2B). At 30 mM HCO_3_^−^, we detected no net increase in methane between day 10 and 12, suggesting that methanogens, like dechlorinators, were also experiencing H_2_ limitation. Once H_2_ became available after the second addition, methane production rates quickly increased in all cultures (Figure 2A).

Upon the third addition of H_2_ (day 26), methane no longer increased at 2.5 mM HCO_3_^−^ even though H_2_ was provided (Figure 2A, day 26–32), indicating a HCO_3_^−^, and not a H_2_ limitation. Even though HCO_3_^−^ was not measured due to analytical limitations, we were able to track HCO_3_^−^ consumption *via* production of methane and acetate, as illustrated in Additional file 1. The HCO_3_^−^ utilization balance presented in Additional file 1 shows that production of methane (and to a lesser degree acetate) exhausted all the HCO_3_^−^ in the systems initially supplemented with 2.5 mM.

Homoacetogenesis exhibited the opposite trend to methanogenesis. According to the time course concentrations recorded and shown in Figure 2C, more acetate was produced when more HCO_3_^−^ buffer was present. Additionally, among all conditions tested, the greatest increase in copies L^−1^ culture by day 7 of the formyltetrahydrofolate synthase (FTHFS) gene, a functional marker for acetogens, was detected at 30 mM HCO_3_^−^ (Figure 2D), and the relative numbers of gene copies were lower with decreasing concentrations of HCO_3_^−^. Before the second addition of H_2_, all cultures showed an increase of 0.3-1.3 mM acetate (Figure 2C). However, after injecting the second dose of H_2_, only a small rise in acetate was observed at 2.5 and 5 mM HCO_3_^−^. In contrast, at 10 and 30 mM HCO_3_^−^, we detected a boost in homoacetogenesis (Figure 2C) and corresponding higher increases in the FTHFS gene (Figure 2D).

The qPCR data for both methanogens and homoacetogens correlate well with our analytical data. The resulting increased levels of homoacetogens coupled to the lowest levels of methanogens at 30 mM HCO_3_^−^ indicate benefits for the first group at the higher HCO_3_^−^ concentrations. Unlike homoacetogens, the resulting methanogenic microorganisms were present at similar levels in cultures initially containing 2.5 and 5 mM HCO_3_^−^ and less plentiful in cultures initially containing 10 and 30 mM HCO_3_^−^ (Figure 2B). Overall, our findings are consistent with the lower HCO_3_^−^ requirement for methane production: one mol HCO_3_^−^ consumed for one mol methane (Equation 1) vs. two mol HCO_3_^−^ consumed for one mol acetate (Equation 2). Additionally, these data are in agreement with the findings of Florencio et al., 1995 [35] on substrate competition between methylotrophic methanogens and methanol-utilizing acetogens in UASB reactors, where acetogenesis was significant and outcompeted methanogenesis only in the presence of exogenously supplemented HCO_3_^−^.

### Distribution of electrons for H_2_-consuming processes

**Figure 3:**
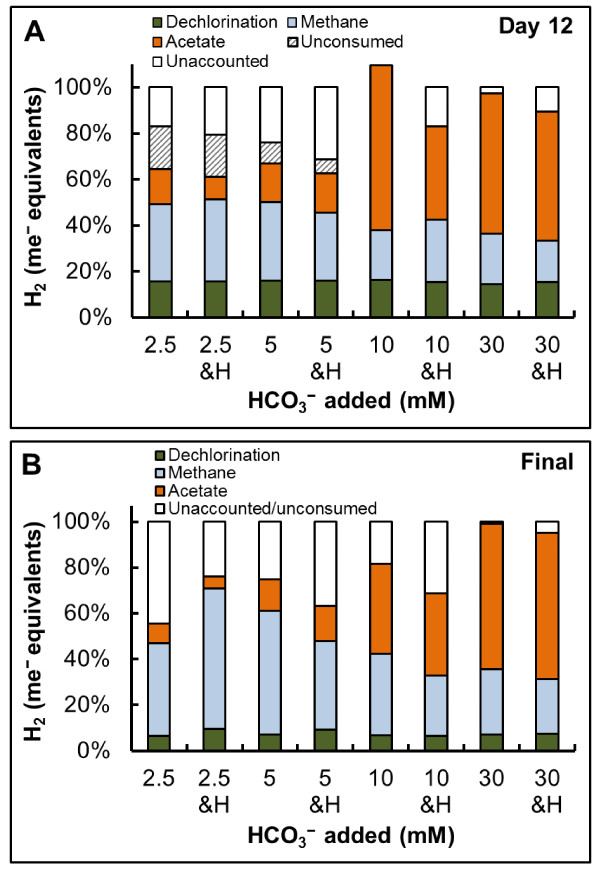
**Distribution of electrons fed as H**_**2 **_**towards dechlorination, methanogenesis, and homoacetogenesis at various HCO**_**3**_^**− **^**concentrations.** (**A**) Average data from triplicate cultures on day 12 after addition of 16.4 me^-^ equiv. H_2_ (8.2 mmol L^−1^)_._ (**B**) Average final data from triplicate cultures after addition of 33 me^-^ equivalents (16.4 mmol L^−1^ H_2_ in 2.5 mM HCO_3_^−^ & HEPES and 5 mM HCO_3_^−^ & HEPES) and 49 me^-^ equivalents (32.8 mmol L^−1^ in all other sets). The presence of the additional buffer, HEPES, is denoted as H on the X-axis.

The fate of electrons fed as H_2_ is depicted in Figure 3. By day 12 (after one addition of H_2_; Figure 3A), 70% or greater of the total added electrons can be accounted for towards the three main energy-deriving reactions, dechlorination, homoacetogenesis and methanogenesis, under all conditions tested. Biomass was not included in these balances, however, a 10-20% fraction of the total electrons can be assumed for cell synthesis [36]. 1.65 mmol H_2,_ the theoretical H_2_ requirement for dechlorination of 0.55 mmol TCE, equals to 3.3 me^-^ equivalents H_2_, and each 8.2 mmol L^–1^ H_2_ addition represents 16.4 me^-^ equivalents. Out of the three main processes occurring in our test systems, TCE dechlorination utilized a small fraction of 9.3% out of the total me^-^ equivalents for the cultures that completed dechlorination with two H_2_ additions (Figure 1B and D), and 6.7% of the total me^-^ equivalents for those that received three H_2_ additions (Figure 1A, C, E, F, G, and H).

From the H_2_ me^-^ equivalents provided at time 0, only 18.3% would have been required to completely reduce TCE to ethene. As seen in Figures 1 and 3A, none of the cultures, regardless of their H_2_ demand, completed dechlorination with the initial H_2_. Additionally, the 10 and 30 mM HCO_3_^−^ amendments with or without HEPES received H_2_ fifteen times in excess of the theoretical demand for dechlorination, yet only the sets supplemented with HEPES completed dechlorination, implicating an important pH factor, which is discussed in the next section.

Overall, the fate of most H_2_ me^-^ equivalents was to HCO_3_^−^-driven reactions towards the production of methane and acetate. Acetate from hydrogenotrophic homoacetogenesis was also found to be the main sink of electrons in a field study that used H_2_ gas for remediation of chlorinated ethenes in groundwater [9]. Moreover, Duhamel and Edwards 2007 [18] investigated the growth and yields of hydrogenotrophic methanogens, acetogens and dechlorinators during the process of dechlorination. The authors found that most of the electrons fed as methanol in 30 mM HCO_3_^-^ buffered medium went towards acetogenesis and, that methanogens were outcompeted by acetogens. Our data from 10 and 30 mM HCO_3_^−^ corroborate their findings; however, one important additional finding from our experiments, as seen in Figure 2 and 3, is that methanogens can outcompete homoacetogens at low HCO_3_^−^ concentrations (2.5 and 5 mM).

The results on TCE dechlorination, methanogenesis and homoacetogenesis from this work at different HCO_3_^−^ concentrations offer some insights into which competing microbial groups will prevail and how HCO_3_^−^ consumption affects rates of dechlorination. Furthermore, our study also alludes to how HCO_3_^−^ drives the H_2_ competition between organohalide respirers, methanogens, and homoacetogens. This important aspect has not been determined previously in reductive dechlorination, to our knowledge. In addition, for application purposes, it is important to consider how temperature could affect these findings, as these predictions might be somewhat different at lower temperatures, such as those in groundwater. Our experiments were performed at 30°C, however, temperature studies on organohalide respirers (i.e. *Dehalococcoides*) have documented slower rates of dechlorination at 10-15°C compared to their maximum rates at 30-35°C [37]. Homoacetogens are even greater H_2_ and HCO_3_^−^ consumers than methanogens at lower temperatures [34,38], hence, the predominance of homoacetogens would be greater in groundwater systems. Furthermore, because many homoacetogens can consume fermentables and/or H_2_ to produce acetate [29], it is important to consider homoacetogenesis as an electron sink and alkalinity-consuming process in dechlorination at the laboratory and field scale. Although comprehensive models on *in situ* reductive dechlorination have been developed [13,14,22,39], the introduction of hydrogenotrophic homoacetogenesis in these models has not been considered.

### Effect of pH on dechlorination in HCO_3_^−^ -amended cultures

**Figure 4:**
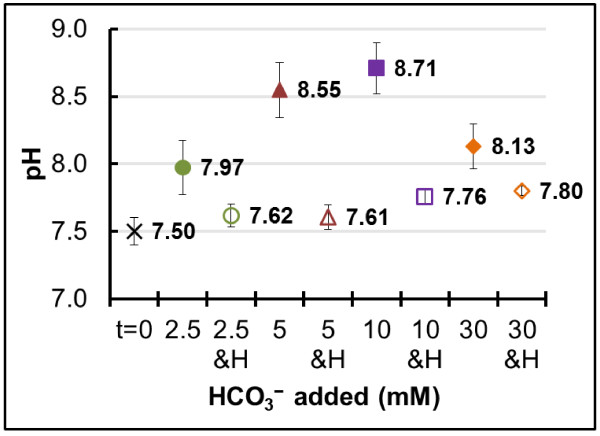
**pH changes resulting from biological HCO**_**3**_^**− **^**consumption.** Average initial (t = 0) and final pH measurements in all HCO_3_^−^ amendments from this study in the absence (closed symbols) or presence (open symbols) of HEPES. The error bars are standard deviations of triplicate cultures. The buffer HEPES is denoted as H on the X-axis.

We supplemented HEPES to all HCO_3_^−^ concentrations tested to separate between the effect of HCO_3_^−^ as an electron acceptor/sink and the effect of pH changes resulting from microbial processes that use HCO_3_^−^ as an electron acceptor, i.e. methanogenesis and homoacetogenesis. The time course pH measurements presented in Additional file 2 and the final measurements in Figure 4 uncovered a trend when HCO_3_^−^ was the sole buffer: a higher pH increase with increasing HCO_3_^−^ concentrations due to methanogenesis and homoacetogenesis HCO_3_^−^-consuming reactions. This was not the case at 30 mM HCO_3_^−^, where we recorded a lower final pH than at 10 mM HCO_3_^−^ (Figure 4) due to the buffering capacity from the remaining 20 mM unconsumed HCO_3_^−^ (Additional file 1). However, in a separate experiment where we increased the total concentration of H_2_ to 41.2 mmol L^−1^ in cultures containing 30 mM HCO_3_^−^, we recorded a final pH of 9.6 under these conditions (data not shown). These cultures also exhibited slower rates of dechlorination compared to the data from Figure 1 and no ethene formed by day 40 of the experiments (data not shown).

An increase in pH at all HCO_3_^−^ concentrations tested was also observed when HEPES was present as an additional buffer but the pH increase was within a much narrower range (Figure 4). We ran statistical analyses and determined that, because of better pH buffering, the rates of dechlorination were significantly faster (Student *t*-test, P <0.05) in the presence of HEPES, compared to when HCO_3_^–^ was the sole buffer (Figure 1). In this study, we show that high pH can also occur in dechlorinating systems, especially in engineered systems fed with H_2_, and this pH change can negatively impact chloroethenes reduction. A detrimental effect on TCE dechlorination that resulted in accumulation of mainly *cis*-DCE at pH 8.3 was previously observed in an anaerobic biotrickling filter [40]. Our results show that high pH is stressful to TCE dechlorinating microorganisms, hence, research on bioremediation of chloroethenes will greatly benefit from comprehensive pH studies.

## Conclusions

Despite the fact that HCO_3_^−^ is a common natural buffer and addition of more HCO_3_^−^ can counteract pH deviations from the optimum range for dechlorination, the results of our study point out that 1) high HCO_3_^−^ concentrations increase the H_2_ demand, and that 2) consumption of HCO_3_^−^ contributes to pH increases that could adversely affect TCE dechlorination rates or result in accumulation of toxic intermediate by-products (i.e., DCE and VC). Our findings regarding the effect of pH increases from HCO_3_^−^-consuming reactions are relevant for *ex situ* chloroethenes remediation technologies that provide H_2_ and for laboratory amendments. When fermentable substrates are used to stimulate reductive dechlorination, or, in the case of groundwater where HCO_3_^−^ is replenished from minerals dissolution or organics oxidation, this increase in pH will likely be offset by the protons produced from fermentation or by the constant supply of buffer.

However, the lessons learned from this study on dechlorination, methanogenesis, and homoacetogenesis highlight that HCO_3_^−^, especially when abundant, could be an important variable for biologically-driven TCE dechlorination, as it has a prominent role as an electron acceptor by stimulating competing H_2_-consuming processes. Our findings also point out that a shift in the main H_2_ competitors occurs depending on the HCO_3_^−^ concentration available in the environment, with homoacetogens as the greater electron sink at high HCO_3_^−^, and methanogens as the main H_2_ competitors at low HCO_3_^−^.

## Methods

### Microbial inoculum and preparation of batch cultures

The sediment-free microbial consortium, DehaloR^2, described by Ziv-El et al., 2011 [20] was used as the inoculum. For the experiments in this study, we preconditioned the inoculum culture by growing it in 10 mM HCO_3_^−^ medium, with excess H_2_ as electron donor, and two consecutive feedings of 10 μL neat TCE in 120 mL medium.

Reduced anaerobic mineral medium was prepared containing the following reagents per liter: 1 g NaCl, 0.06 g MgCl_2_ × 6H_2_O, 0.2 g KH_2_PO_4_, 0.3 g NH_4_Cl, 0.3 g KCl, 0.005 g CaCl_2_ × 2H_2_O, and 1 mL of Trace A and Trace B solutions described elsewhere [17]. During medium preparation, nitrogen was the sole gas for boiling and bottling and the reducing agents were 0.2 mM L-cysteine and 0.2 mM Na_2_S × 9 H_2_O. No buffer was added to the medium before autoclaving. For bottling, we used 160-mL glass serum bottles containing 90 mL liquid and 70 mL headspace sealed with black butyl rubber stoppers and aluminum crimps.

The concentrations of NaHCO_3_ tested were 2.5, 5, 10, and 30 mM. In the cultures where both NaHCO_3_ and HEPES (pK_a_ = 7.55) were used as buffers, we supplemented 5 mM HEPES in combination with 2.5, 5 and 10 mM HCO_3_^−^, and 10 mM HEPES in the 30 mM HCO_3_^−^ cultures. NaHCO_3_ and HEPES were delivered to each bottle from 1 M sterile anaerobic stock solutions. The initial pH was adjusted with sterile 2.25 N HCl or NaOH to 7.5 (± 0.1). At time 0, we added to each culture bottle 0.55 mmol L^–1^ TCE (5 μL neat or 71.3 mg L^−1^), 1 mL ATCC vitamin mix, 50 μL of 1 g L^−1^ vitamin B_12_ solution, 8.2 mmol L^–1^ H_2_ (20 mL H_2_ gas), and 10 mL DehaloR^2 microbial culture, corresponding to a 10% inoculum. The working volume was 100 mL of liquid with 60 mL of headspace. The bottles were incubated in the dark at 30°C without shaking. An additional 8.2 mmol L^–1^ H_2_ was added on day 12 (all cultures) and on day 26 (only to cultures still undergoing dechlorination). All experimental conditions were tested in triplicates and the experiments were also performed on two separate occasions.

### Chemical and pH measurements for the time course experiments

We measured TCE, *cis*-DCE, VC, ethene, and methane using a gas chromatograph (GC) (Shimadzu GC-2010; Columbia, MD) equipped with a flame ionization detector (FID). The compounds were carried by helium gas through an Rt-QS-BOND capillary column (Restek; Bellefonte, PA). The oven temperature was maintained at 110°C for 1 min, followed by a temperature increase of 50°C min^−1^ to 200°C. Then, the temperature ramp was further raised to 240°C with a 15°C min^−1^ gradient and held for 1.5 mins. The temperatures of the FID and the injector were 240°C. Chloroethenes, ethene and methane calibrations in 160-mL bottles with 100 mL liquid volume were performed in a range of 0.05-2.45 mmol L^–1^. The detection limit for all compounds measured on the GC-FID is ≤0.018 mmol L^–1^.

A GC instrument equipped with a thermal conductivity detector (TCD) was employed to measure H_2_ before reinjecting additional H_2_ to the cultures on day 12. The instrument settings used were those previously outlined [41]. The H_2_ detection limit for the GC-TCD is 0.8% vol/vol.

We quantified acetate, propionate, and formate from 0.75-mL liquid samples filtered through a 0.2 μm polyvinylidene fluoride membrane syringe filter (Pall Corporation; Ann Arbor, MI) into 2-mL glass vials (VWR; Radnor, PA) *via* high performance liquid chromatography (HPLC) using a previously published method [41]. Five point calibration curves (0.5-10 mM) were generated for acetate, propionate, and formate during every HPLC run. The detection limit for the compounds measured on the HPLC was ≤0.1 mM.

0.29 ± 0.06 mM propionate was carried over from the inoculum culture and the final measured concentration was 0.33 ± 0.04 mM, indicative that propionate did not serve as a significant source of electrons. Formate was sometimes also detected at low concentrations (0.1-0.3 mM), however, we did not identify a clear trend on the formation/consumption of this product. Therefore, propionate and formate were omitted from the electron balances in Figure 3.

The pH was measured using an Orion 2-Star pH bench top meter (Thermo Scientific, USA) that was calibrated regularly with 4.01, 7.00, and 10.01 standard solutions from the manufacturer.

All cultures were sampled for gas and liquid analyses until dechlorination of TCE to ethene was complete or until the end of experiments on day 40.

### DNA extraction and molecular microbial characterization

Pellets were formed by centrifugation from 2-mL liquid from each culture replicate and they were stored at −20°C until the DNA extraction. Genomic DNA was extracted for two time points for all sets of HCO_3_^−^ & HEPES, and two time points for the set with 30 mM HCO_3_^−^ only. Before DNA extraction, the replicate pellets were thawed, resuspended in the supernatant, and combined, so that only extraction per set per time point was performed. This was done to increase total biomass and DNA yield. The DNA extraction was performed as previously described [20].

We employed quantitative real-time PCR to target the 16S rRNA gene of *Dehalococcoides* and *Archaea* (TaqMan® assays) and the FTHFS gene of homoacetogens (SYBR Green assay). Triplicate reactions were setup for the six point standard curves and the samples in 10 μL total volume using 4 μL of 1/10 diluted DNA as template. We generated standard curves by serially diluting 10 ng μL^–1^ plasmid DNA. The primers and probes, reagents concentrations, and thermocycler (Realplex 4S thermocycler; Eppendorf, USA) conditions were those described for *Dehalococcoides*[42], *Archaea*[43,44], and FTHFS [44,45]. Acetoclastic methanogens (the order *Methanosarcinales*) were not assayed because they are absent in the culture employed in this study, which this was confirmed by qPCR previously [20].

Time 0 for all qPCR assays was generated by amplifying genomic DNA from the inoculum culture and assigning 10% as the starting concentrations of gene copies per L culture.

### Calculations

The distributions of electrons from Figure 3 were calculated in units of me^-^ equivalents for each compound from the equation below:

(3) $$\%\text{compound=}\frac{\left[\text{compound}\right]\times \frac{\text{\#electrons}}{\text{mol}}}{\left[{\text{H}}_{2}\right]\times \frac{2\;\text{electrons}}{{\text{mol H}}_{2}}}\times 100$$

The number of me^-^ equivalents for dechlorination is 2, 4, and 6 for DCE, VC and ethene, respectively, 8 for acetate and methane, and 2 for H_2_.

## End notes

^a^Throughout this work, HCO_3_^−^ is used to denote the buffer HCO_3_^−^/CO_2_. At the pH ranges observed in this study, HCO_3_^−^ accounted for 90% or greater of the two species.

## Competing interests

The authors declare that they have no competing interests.

## Authors’ contributions

AGD and RKB designed the experiments that led to the writing of the manuscript. AGD and DFW carried out the experimental work. PP participated in the performance and analyses for quantitative PCR. AGD drafted the manuscript; RKB, PP, and RUH critically reviewed and contributed to the intellectual merit of the paper. All authors read and approved the final version of the manuscript.

## Supplementary Material

Additional file 1 **Calculated HCO**_**3**_^**− **^**consumption for methane and acetate production.** Click here for file

Additional file 2 Time course pH measurements. Click here for file
