# Supplementary material for: Role of bicarbonate as a pH buffer and electron sink in microbial dechlorination of chloroethenes
Source: Microb Cell Fact. 2012 Sep 13;11:128. doi: 10.1186/1475-2859-11-128 (PMC3511292; doi:10.1186/1475-2859-11-128)
Supplement: Additional file 1 — Calculated HCO3− consumption for methane and acetate production. [file 1475-2859-11-128-S1.pdf]

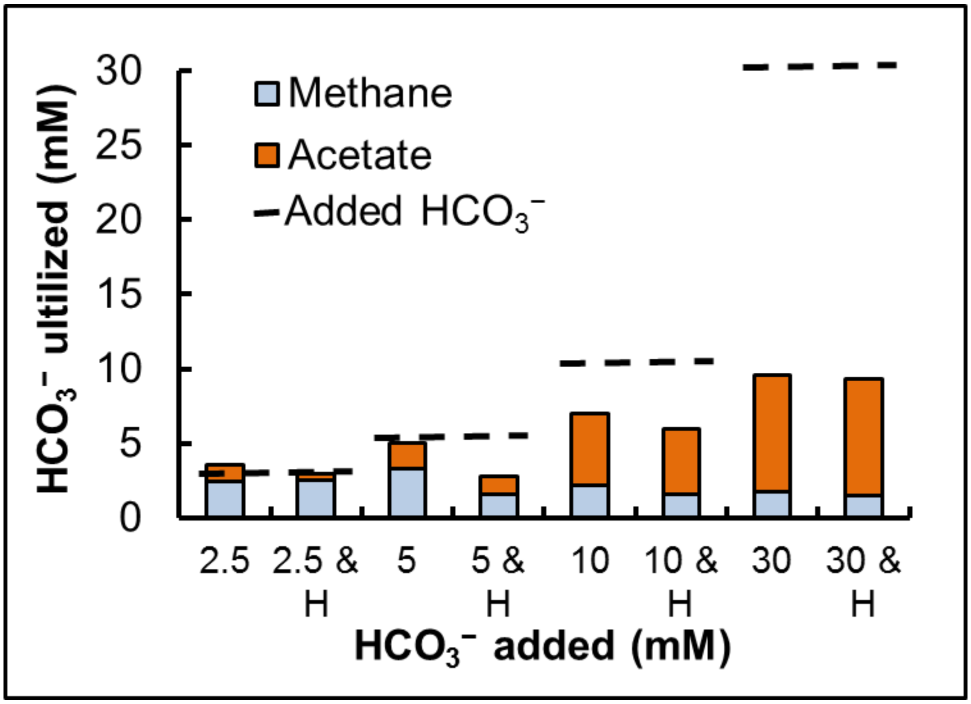

Additional file 1 – Calculated HCO_3^- consumption for methane and acetate production

Calculated HCO_3^- utilization by hydrogenotrophic methanogens and hydrogenotrophic homoacetogens at the end of the experiments in the absence or presence of HEPES (denoted as H on the X-axis). A maximum of 1 mM HCO_3^- was assumed as carryover from the 10% inoculum culture, which was grown in 10 mM HCO_3^- medium. The stoichiometric requirement for methane is one HCO_3^- and for acetate is two HCO_3^-.
