# Supplementary material for: Role of bicarbonate as a pH buffer and electron sink in microbial dechlorination of chloroethenes
Source: Microb Cell Fact. 2012 Sep 13;11:128. doi: 10.1186/1475-2859-11-128 (PMC3511292; doi:10.1186/1475-2859-11-128)
Supplement: Additional file 2 — Time course pH measurements. [file 1475-2859-11-128-S2.pdf]

Additional file 2 – Time course pH measurements

Average pH values with standard deviations of triplicate cultures containing 2.5, 5, 10, and 30 mM HCO_3^- as the sole buffer and a combination of HCO_3^- and HEPES. The values in bold are the final pH measurements.

[HCO_3^-] mM

Day	2.5	2.5 & HEPES	5	5 & HEPES	10	10 & HEPES	30	30 & HEPES
0	7.49 ± 0.06	7.48 ± 0.08	7.47 ± 0.05	7.51 ± 0.07	7.54 ± 0.05	7.46 ± 0.04	7.44 ± 0.03	7.43 ± 0.03
1	7.41 ± 0.07	7.43 ± 0.08	7.45 ± 0.07	7.51 ± 0.04	7.56 ± 0.03	7.45 ± 0.06	7.45 ± 0.09	7.50 ± 0.02
7	7.23 ± 0.05	7.38 ± 0.09	7.37 ± 0.06	7.44 ± 0.09	7.66 ± 0.18	7.47 ± 0.06	7.56 ± 0.07	7.58 ± 0.03
17	7.29 ± 0.03	7.26 ± 0.12	7.36 ± 0.03	7.48 ± 0.12	7.87 ± 0.11	7.53 ± 0.04	7.60 ± 0.11	7.60 ± 0.03
19	–	7.58 ± 0.01	–	**7.61 ± 0.09**	–	–	–	–
20	–	–	–		–	–	–	–
23	7.81 ± 0.06	–	7.92 ± 0.09		8.12 ± 0.11	–	–	–
26	–	**7.62 ± 0.09**	–		–	7.64 ± 0.01	7.68 ± 0.09	7.65 ± 0.01
28	–		–		–	**7.76 ± 0.05**	–	–
32	**7.97** ± 0.20		–		–		–	**7.80 ± 0.04**
35			**8.55 ± 0.20**		8.73 ± 0.10		8.07 ± 0.19	
40					**8.71 ± 0.19**		**8.13 ± 0.17**	
